# Severe depression when everything else is dismissed. A case

**DOI:** 10.1192/j.eurpsy.2021.1623

**Published:** 2021-08-13

**Authors:** L. Soldado Rodriguez, S.S. Sánchez Rus, A. Alvarado Dafonte

**Affiliations:** 1 Mental Health Unit, Complejo Hospitalario de Jaen, Jaen, Spain; 2 Jaén, Complejo Hospitalario Jaén, Jaén, Spain

**Keywords:** Hospitalization, Simulation, factitius disorder, mmpi-2

## Abstract

**Introduction:**

Simulation is a deliberate counterfeiting of physical or psychological symptoms in order to obtain a secondary gain or external incentive, like evading from military service, scape from work, obtain economic compensations or avoid criminal responsibility. It is estimated that prevalence is roughly 1% in mental health patients, with higher prevalence in young males and middle aged. Male with 52 years attends to emergency service. Erratic tracking in Mental Health Service from two years ago with unfavorable progress. He goes to emergency service referring aggravation of discomforted state of mind even with readjustment a week ago. Addiction to benzodiazepines and clinophilia. Currently with temporary inability to work of large data.

**Objectives:**

To set a differential diagnose between depression, factitious disorder and malingering.

**Methods:**

Examination shows moderated sad mood with despair, reactive to disability and progression of his illness. Sparing in words speech, focused on life or work problems. Autolytic verbalizations and self-control inability.

**Results:**

Mmpi2 that shows: Gough’s F-K. Dissimulation index, 34. Cut-off point to consider simulation/ pretending being ill varies among authors. A conservative cut-off point is 15, showing a severe exaggeration of its discomfort and dissimulation.

**Conclusions:**

It is important to make an appropriate anamnesis and psychopathological exploration, as well as observation to reach a correct diagnose. In this case, clearly secondary gain was founded, therefore diagnose was malingering.

**Disclosure:**

No significant relationships.

